# Experimental Evolution of an RNA Virus in Wild Birds: Evidence for Host-Dependent Impacts on Population Structure and Competitive Fitness

**DOI:** 10.1371/journal.ppat.1004874

**Published:** 2015-05-20

**Authors:** Nathan D. Grubaugh, Darci R. Smith, Doug E. Brackney, Angela M. Bosco-Lauth, Joseph R. Fauver, Corey L. Campbell, Todd A. Felix, Hannah Romo, Nisha K. Duggal, Elizabeth A. Dietrich, Tyler Eike, Jennifer E. Beane, Richard A. Bowen, William C. Black, Aaron C. Brault, Gregory D. Ebel

**Affiliations:** 1 Department of Microbiology, Immunology and Pathology, College of Veterinary Medicine and Biomedical Sciences, Colorado State University, Fort Collins, Colorado, United States of America; 2 United States Department of Agriculture, Animal and Plant Health Inspection Service, Wildlife Services, Lakewood, Colorado, United States of America; 3 Division of Vector-Borne Diseases, Centers for Disease Control and Prevention, Fort Collins, Colorado, United States of America; 4 Section for Computational Biomedicine, Boston University School of Medicine, Boston, Massachusetts, United States of America; 5 Department of Biomedical Sciences, College of Veterinary Medicine and Biomedical Sciences, Colorado State University, Fort Collins, Colorado, United States of America; Institut Pasteur, FRANCE

## Abstract

Within hosts, RNA viruses form populations that are genetically and phenotypically complex. Heterogeneity in RNA virus genomes arises due to error-prone replication and is reduced by stochastic and selective mechanisms that are incompletely understood. Defining how natural selection shapes RNA virus populations is critical because it can inform treatment paradigms and enhance control efforts. We allowed West Nile virus (WNV) to replicate in wild-caught American crows, house sparrows and American robins to assess how natural selection shapes RNA virus populations in ecologically relevant hosts that differ in susceptibility to virus-induced mortality. After five sequential passages in each bird species, we examined the phenotype and population diversity of WNV through fitness competition assays and next generation sequencing. We demonstrate that fitness gains occur in a species-specific manner, with the greatest replicative fitness gains in robin-passaged WNV and the least in WNV passaged in crows. Sequencing data revealed that intrahost WNV populations were strongly influenced by purifying selection and the overall complexity of the viral populations was similar among passaged hosts. However, the selective pressures that control WNV populations seem to be bird species-dependent. Specifically, crow-passaged WNV populations contained the most unique mutations (~1.7× more than sparrows, ~3.4× more than robins) and defective genomes (~1.4× greater than sparrows, ~2.7× greater than robins), but the lowest average mutation frequency (about equal to sparrows, ~2.6× lower than robins). Therefore, our data suggest that WNV replication in the most disease-susceptible bird species is positively associated with virus mutational tolerance, likely via complementation, and negatively associated with the strength of selection. These differences in genetic composition most likely have distinct phenotypic consequences for the virus populations. Taken together, these results reveal important insights into how different hosts may contribute to the emergence of RNA viruses.

## Author Summary

Viruses are constantly emerging into new areas and pose significant challenges to public health. Chikungunya and West Nile viruses (WNV), both mosquito-borne RNA viruses, are quintessential examples of how increased globalization has facilitated the expansion of viruses into new territories. Rapid evolution of both of these agents has contributed to their rapid spread and health burden. Thus, characterizing how selection shapes zoonotic RNA viruses in their natural hosts is important to understand their emergence. As an ecological generalist able to infect hundreds of bird species, WNV is an excellent tool to study how different animal hosts can differentially drive virus evolution. We examined the genetic composition and fitness of WNV produced during replication in wild-caught American crows, house sparrows and American robins, species that range in mortality following WNV infection (crows the highest, robins the lowest). We demonstrate host-dependent effects on WNV population structure and fitness. Our study provides insights on how different virus-animal interactions can influence the success of a virus in the next host and ultimately the success of virus emergence into new host systems.

## Introduction

RNA viruses pose some of the most complex, persistent and challenging problems facing public health and medicine. The ongoing outbreaks of avian influenza A(H7N9) virus (*Orthomyxoviridae*) in China [1], Ebola virus (*Filoviridae*) in West Africa [2], and chikungunya virus (CHIKV, *Togaviridae*, *Alphavirus*) and West Nile virus (WNV, *Flaviviridae*, *Flavivirus*) in the Americas [3,4] highlight the health and societal impacts imposed by RNA virus-induced diseases. Several factors contribute to the emergence of these agents and the continued burdens they impose on human health. Among these is their ability to undergo rapid evolution in new and/or changing environments. Well documented examples of RNA virus evolution leading to increased virus transmission include WNV and CHIKV. In both cases, small, conservative amino acid substitutions (residues with similar physiochemical properties) to the viral envelope proteins resulted in more efficient transmission by mosquito vectors [5,6]. Adaptive changes to RNA virus genomes first arise as minority components within a genetically complex population of related but non-identical virus variants. The genetic diversity present in naturally occurring RNA virus populations has been clearly shown through a large and expanding body of observational and experimental studies to be critical to their biology. For example, several studies have demonstrated that the diversity of an intrahost viral population, rather than the fitness of individual variants, correlates with pathogenesis, disease progression and therapeutic outcome [7–9]. Moreover RNA viruses have the capacity for rapid evolutionary change because within infected hosts, all single nucleotide mutations may be generated.

This has been particularly clear in the case of WNV, an arthropod-borne virus (arbovirus) that persists in nature in enzootic cycles between ornithophilic mosquitoes (mainly *Culex* spp.) and birds. After its initial identification in the New York City area in 1999, WNV spread throughout the continental United States, producing the largest outbreaks of flaviviral encephalitis ever recorded in North America. The explosive spread of the virus was accompanied by the displacement of the introduced genotype by a derived strain that is more efficiently transmitted by local *Culex* mosquitoes [10]. Studies of intrahost population dynamics of WNV demonstrated that genetic diversity is greater in mosquitoes than in birds [11]. The selective basis for the host-specific patterns of WNV genetic diversity is that the strong purifying selection that predominates in birds is relaxed in mosquitoes [11,12]. In addition, the RNA interference-based antiviral response in mosquitoes creates an environment where negative frequency-dependent selection may drive rare variants to higher population frequency [13]. Moreover, WNV maintains both adaptive plasticity and high fitness by alternating between hosts that impose different selective forces on the virus population [14].

Nonetheless, important gaps remain in our understanding of how error-prone replication interacts with selective and stochastic reductions in viral genetic diversity under natural conditions. This is particularly the case for arboviruses, which tend to cause acute infection in vertebrates, with transmission occurring before the development of a neutralizing antibody response. Therefore, well-described mechanisms of immune selection such as those that occur during chronic hepatitis C and human immunodeficiency virus infections are comparatively weak during acute arbovirus infection of vertebrates. Thus, the ways that ecologically relevant, natural hosts can influence arbovirus genetic diversity remain poorly understood. WNV in particular provides an excellent experimental system to study the influences of natural vertebrate hosts on viral evolution. The virus infects a large number of wild bird species [15] with a wide-range of infection outcomes [16]. In addition, several studies have provided evidence that particular WNV variants may arise through adaptation to birds [17,18].

Therefore, we sought to determine whether different wild bird species may have distinct impacts on WNV population structure. Specifically, we allowed WNV to replicate in wild-caught American crows (*Corvus brachyrhynchos*), house sparrows (*Passer domesticus*), and American robins (*Turdus migratorius*), bypassing the mosquito portion of the arbovirus cycle in order to focus on the impact of different vertebrate environments on virus populations during acute infection. Virus was passaged in individuals of each species five times in order to amplify host-specific patterns of selection that may remain cryptic after a single passage. Bird species were selected on the basis of ecological relevance and resistance to WNV-induced mortality. American crows experience high viremia and mortality following inoculation with WNV [19] and can directly transmit virus to roost mates without mosquito involvement [20]; house sparrows experience high viremia and intermediate mortality [21] and are frequently involved in WNV perpetuation [22]; and American robins experience intermediate viremia but very low mortality [23] and can be drivers for human WNV risk [24]. Virus populations were characterized using next generation sequencing (NGS) and through *in vivo* fitness competition studies in birds and mosquitoes. Our findings demonstrate that relevant vertebrate hosts with varying levels of disease susceptibility differentially shape WNV population structure with direct impacts on fitness during host shifts.

## Results

### Virus passage and phenotypic assessment

**Fig 1:**
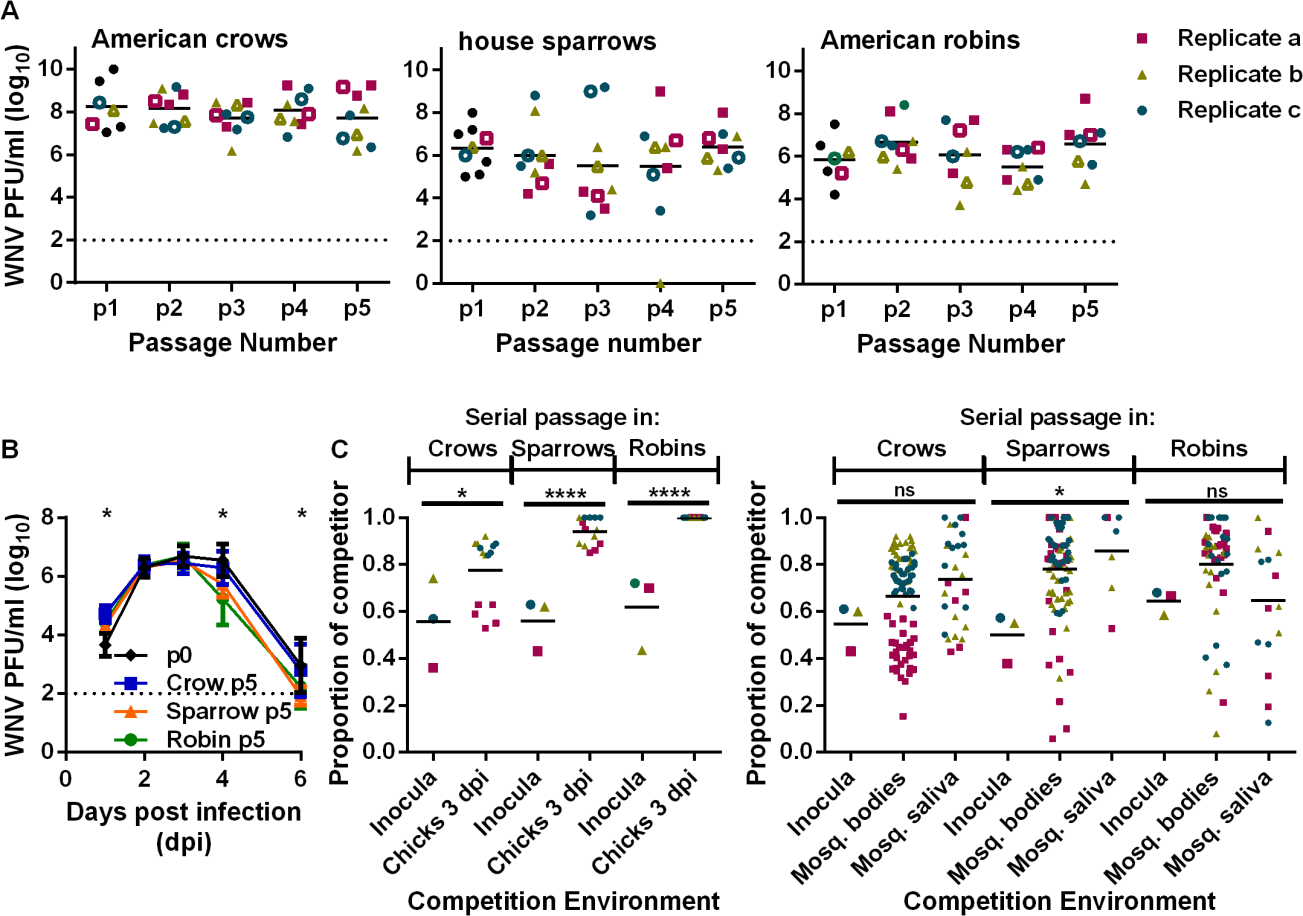
Passage of WNV in birds results in competitive fitness increases while viremia remains unchanged. (**A**) WNV titers during passage. Open symbols represent samples with median viremias that were used for subsequent passage. (**B**) Viremia production after sequential passage, measured in young chickens (mean ± SD, n = 12–15 chickens each, data from passage replicates combined, *, P < 0.01,two-way ANOVA with Tukey’s correction). Dashed lines indicate the assay detection limits. (**C**) Competitive replicative fitness in young chickens (left; *, *P* = 0.0339; ****, *P* < 0.0001, unpaired t-test) and mosquitoes (right; ns, not significant; *, *P* < 0.05, unpaired t-test for both bodies and saliva). Passage replicates are colored as in (A) and horizontal lines represent the mean proportion of bird-passed WNV. Phenotypic assessment of wild bird passaged virus in its passaged host and in orally infected mosquitoes are in S1 Fig.

The WNV used in these studies was derived from an infectious clone of the NY99 genotype and is described in detail elsewhere [25]. Clone-derived WNV was passaged five times in wild-caught American crows, house sparrows and American robins. To avoid systematically selecting high- or low-replicating strains and population bottlenecks during passage, and since titers are highly variable in wild-caught birds, the sera from the individuals with the intermediate viral load were passed into the next cohort at a standard dose of 1000 plaque forming units (PFU). Virus titer was variable but did not change significantly or consistently during the course of passage (Fig 1A). Further, five passages in wild birds did not alter viremia production or mortality in crows and sparrows (S1A and S1B Fig). WNV replication and fitness after passage was assessed using young chickens and *Culex quinquefasciatus* mosquitoes to directly compare the viral populations in hosts not used for passaging and to remove the variability of wild-caught birds (e.g. age and infection history) (Fig 1B and 1C). Passaged virus (p5) was similar to the WNVic (p0) in peak viremia production in chickens (i.e. at 2 and 3 dpi) (Fig 1B).

Fitness assays were used to directly compare passaged viruses to a standard reference WNV in head-to-head competition. These assays can detect subtle fitness differences that are inapparent in comparative studies. Competitive fitness of all wild-bird p5 WNV was significantly enhanced in chickens. Crow-passaged virus had the smallest fitness gains and robin-passaged virus the largest (Fig 1C). Fitness studies conducted in wild birds produced the same results as those in chickens (S1C Fig). Competitive fitness was slightly increased in mosquitoes, but no bird-specific differences were noted (Fig 1C, S1D Fig).

### Patterns of intrahost mutational diversity

At each passage virus was examined by NGS to determine whether the consensus sequence changed during passage and to characterize the diversity of intrahost viral populations (S1 Table, S2 Fig). WNV genome coverage was variable across the genome and between samples (S2A Fig), and positively correlated with viral population size (S2C Fig). The lower relative WNV genome coverage from robin sera can in part be explained by smaller intrahost viral population sizes and smaller virus to host RNA ratios. Approximately 68%, 29% and 7% of NGS reads aligned to the WNV genome from crow, sparrow and robin sera, respectively. Comparatively, 20% and 0.5% of the NGS reads aligned to the WNV genome from chicken sera and mosquito bodies, respectively.

Three nucleotide mutations that led to consensus amino acid substitutions were detected though passaging in birds, but none became fixed (i.e. frequency = 1) in the population. In contrast, three consensus amino acid substitutions were detected after a single mosquito passage. All intrahost single nucleotide variants (iSNVs) > 0.02 frequency are listed in S2 Table.

**Fig 2:**
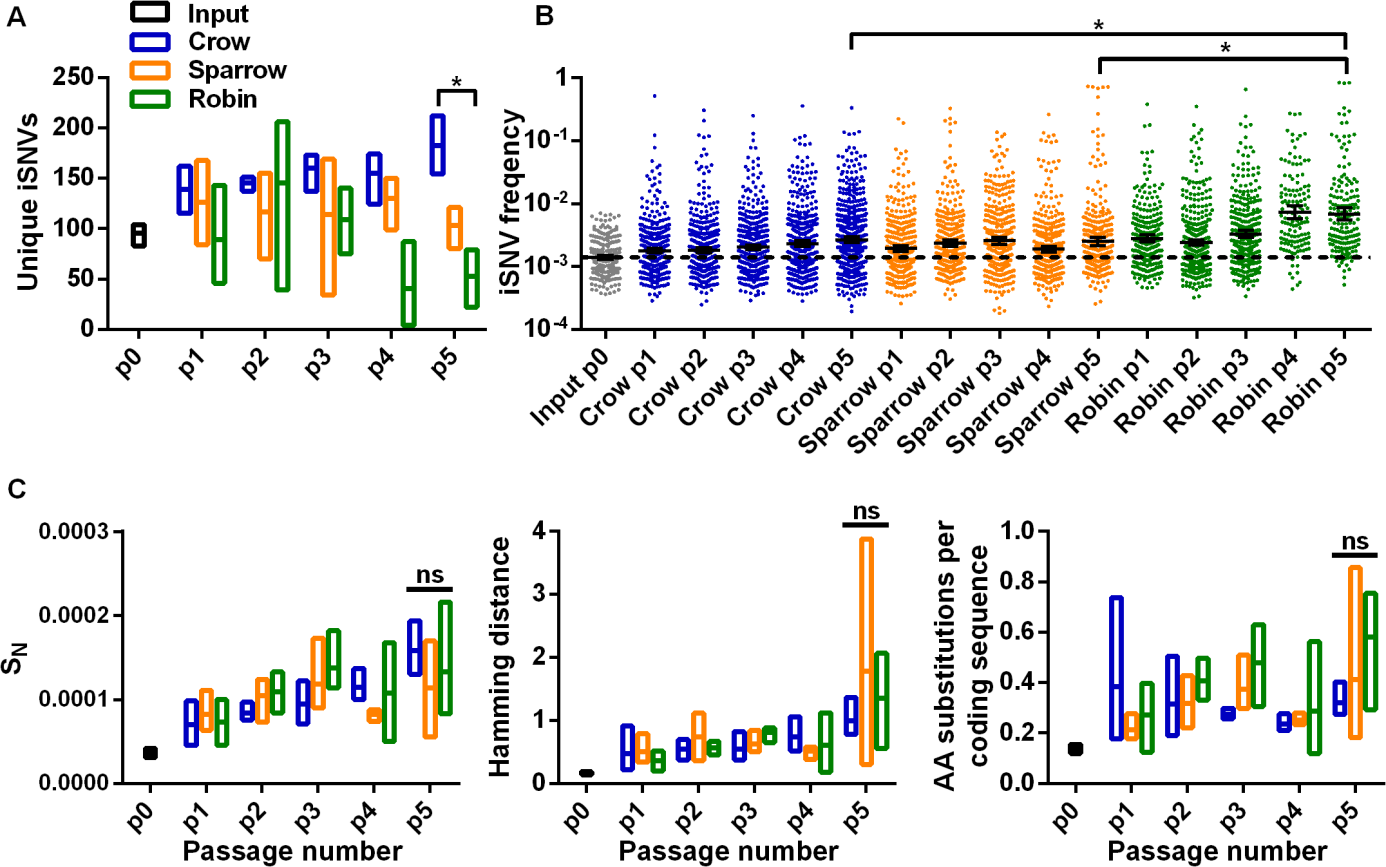
Disparate adaptive routes in birds lead to similar overall intrahost population complexity and diversity. The number (**A**, mean ± range) and frequency (**B**, geometric mean ± 95% CI) of unique intrahost single nucleotide variants (iSNVs,) from the WNV coding sequence during passage in wild-caught crows, sparrows or robins (*, P < 0.05, Kruskal-Wallis test with Dunn’s correction). (**C**) Mean (± range) normalized Shannon entropy (S_N_, measure of population complexity) (left), Hamming distance from the p0 consensus sequence (SNVs per coding sequence) (middle) and the number of amino acid (AA) substitutions per coding sequence (right) (ns, not significant).

We estimated intrahost variation from NGS data to determine whether WNV population diversity was bird species-dependent. The mean number of unique iSNVs in each virus population was relatively constant between passages, but differences were apparent among bird species (Fig 2A). WNV populations passaged in crows five times (p5) had significantly more unique iSNVs than WNV passaged in sparrows and robins. In addition, the frequency of individual iSNVs increased during passage in a species-dependent manner: The mean iSNV frequency after p5 in robins was significantly higher than after p5 in crows or sparrows (Fig 2B). Despite these differences, the viral populations had similar Normalized Shannon entropies (S_N_), Hamming distances (i.e. SNVs per coding sequence) and amino acid substitutions per coding sequence after p5 in different species (Fig 2C).

**Fig 3:**
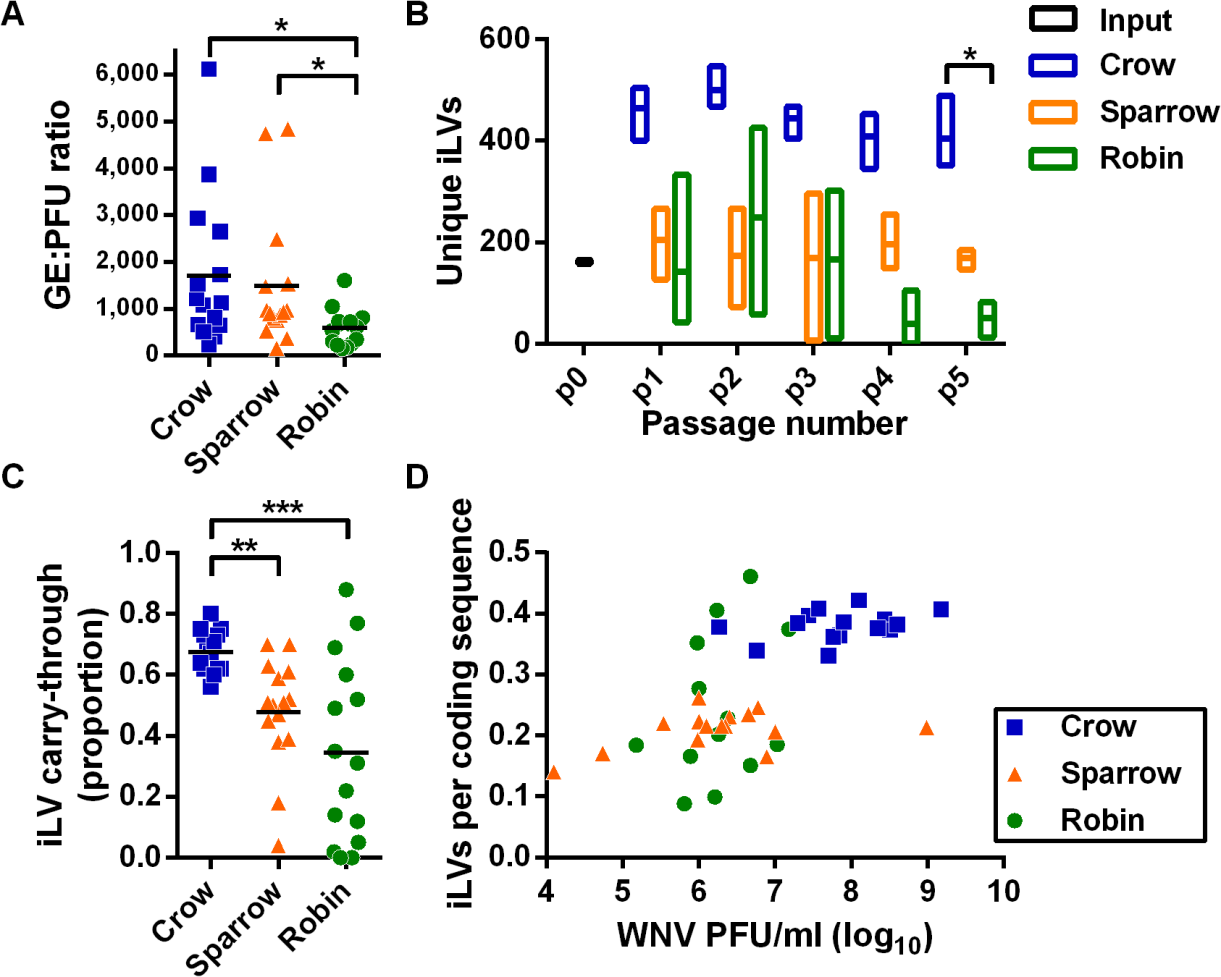
Intrahost virus population density contributes to the accumulation of deleterious mutations. (**A**) WNV genome equivalent to plaque-forming unit (GE:PFU) ratio from all bird passages (n = 15 per species; *, P < 0.05, Kruskal-Wallis test with Dunn’s correction). (**B**) Unique intrahost length variants (iLVs, i.e. single nucleotide insertions and deletions, mean ± range) from the WNV coding sequence (*, crow p5 vs robin p5, *P* = 0.0219, Kruskal-Wallis test with Dunn’s correction) and (**C**) proportion of unique iLVs detected in the subsequent replicate series passages (e.g. carry-through from p1a to p2a) calculated after each passage (**, *P* = 0.0084; ***, *P* = 0.0005, Kruskal-Wallis test with Dunn’s correction). (**D**) Correlation of virus population sizes (PFU/ml) to the number of iLVs per coding sequence from each individual (Pearson r = 0.6150, *P* < 0.001).

We examined the ratio of viral genome equivalents (GE) to PFUs and intrahost single nucleotide length variants (iLVs, including both insertions and deletions) to assess defective viral genomes in WNV populations during passage. Crow-passaged WNV had the highest GE:PFU ratio (Fig 3A) and the most unique iLVs (Fig 3B). In addition, a greater proportion of the iLVs in crows were found in subsequent passages compared to sparrows and robins (Fig 3C). The number of iLVs per coding sequence was positively correlated with the titer of infectious virus (Fig 3D). We then evaluated the possibility that greater levels of iLV carry though in crows, which can only occur via complementation (Fig 3C), were due to sampling artifacts. To do this, we used a hypergeometric test implemented in R that indicated that selecting 400 common iLVs in two samples of 600 from the total pool of available single-nucleotide iLVs (n = 51,490) was 0. Simulation studies confirmed that it is extremely unlikely that random sampling produced the observed data.

### Intrahost selective pressures

Evidence for natural selection was assessed in WNV populations using intrahost neutrality tests. The proportion of mutations in each population that were nonsynonymous (*pN*) and the ratios of nonsynonymous to synonymous variants per site (*d*_*N*_ */d*_*S*_) were highest in the input p0 WNV population and decreased significantly during passage in each bird species (Table 1). Separate analysis of *d*_*N*_ and *d*_*S*_ shows that *d*_*N*_ did not significantly increase during passage while *d*_*S*_ increased significantly at p5 in all bird species, a hallmark of purifying selection. The Fu and Li’s *F* and Fay and Wu’s *H* statistics were obtained from reconstructed haplotypes. The *F* statistic at p1 and p5 was consistently negative, indicating that the haplotypes contained excessive amounts of rare SNVs, again indicative of purifying selection (Table 1). The *H* statistic measures an excess of high compared to intermediate frequency SNVs. The insignificant H values suggest that the deviations from neutrality were due to natural selection rather than selective sweeps (Table 1).

**Table 1:** Intrahost tests of neutrality.

Passage	*pN*	*d*_*N*_ */d*_*S*_	*d*_*N*_	*d*_*S*_	Fu and Li's *F*	Fay and Wu's *H*
p0	0.84	1.57	2 × 10^–5^	1 × 10^–5^	NA	NA
Crow p1	0.80	1.22	5 × 10^–5^	4 × 10^–5^	-0.62029	0.36792
Crow p5	0.35*	0.18*	4 × 10^–5^	3 × 10^–4^ *	-4.17419**	0.77866
Sparrow p1	0.45	0.25	3 × 10^–5^	1 × 10^–4^	-1.83693	0.4298
Sparrow p5	0.33*	0.23*	6 × 10^–5^	6 × 10^–4^ *	-1.16389	1.87529
Robin p1	0.73	0.84	4 × 10^–5^	4 × 10^–5^	-0.83631	0.22204
Robin p5	0.46*	0.25*	9 × 10^–5^	4 × 10^–4^ *	-1.21878	1.85143

*, *P* < 0.05, compared to p0 by the Kruskal-Wallis test with Dunn’s correction for multiple comparisons.

**, *P* < 0.02, critical values compared by two tailed tests in DnaSP.

NA, not applicable because there was only one dominant haplotype.

**Fig 4:**
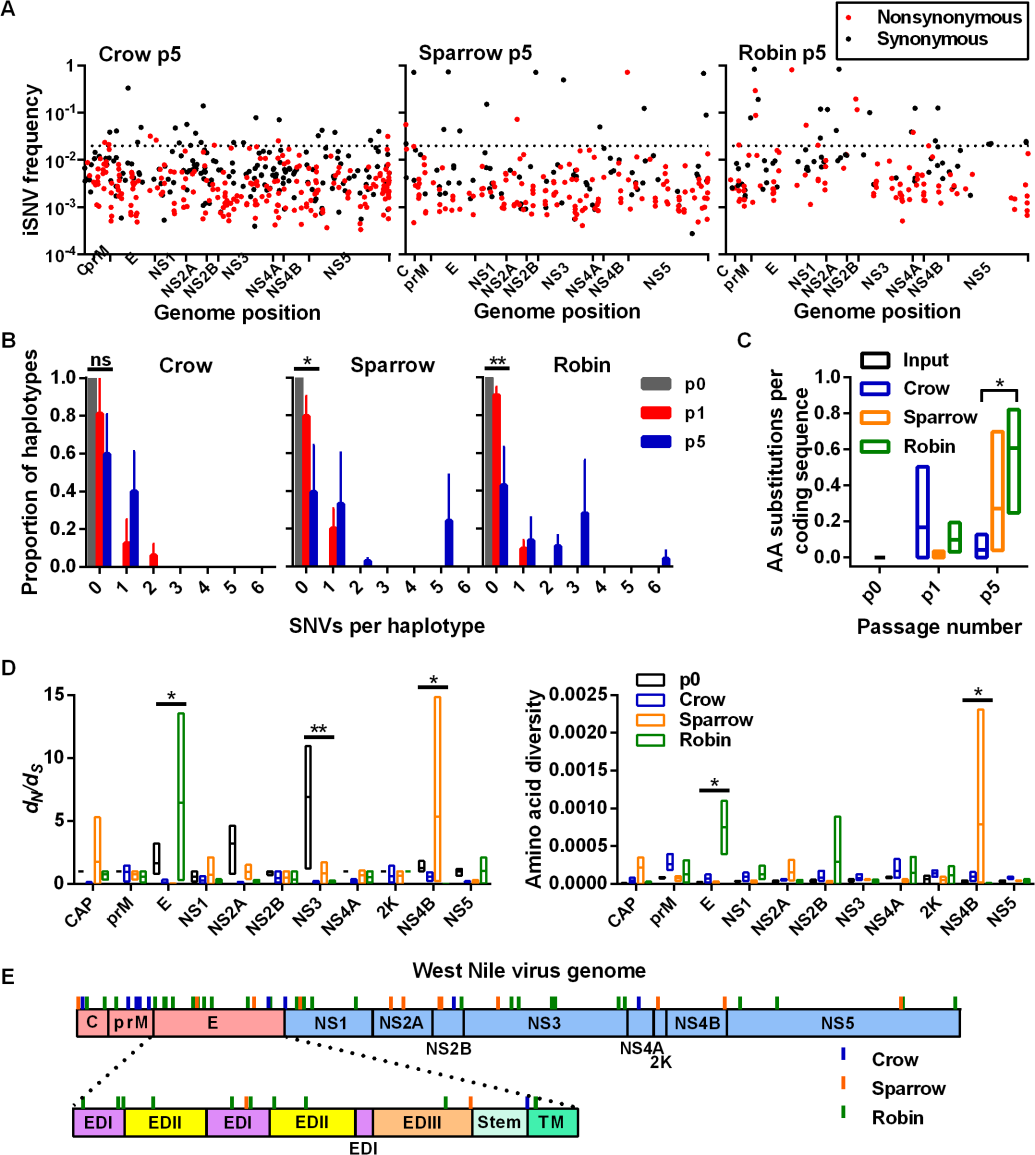
High-frequency iSNVs contribute to haplotype displacement in a bird-species dependent manner. (**A**) iSNVs from input virus (p0) and after passage 5 (p5, all replicates combined) plotted according to genome position. Red and black dots represent synonymous and nonsynonymous iSNVs, respectively. Dotted line represents division between high and low frequency iSNVs (0.02). (**B**) Haplotypes were reconstructed from high frequency iSNVs represented by the number of SNVs per haplotype (i.e. Hamming distance from the p0 haplotype, ± SEM) (ns, not significant; *, *P* = 0.0250; **, *P* = 0.0036, Kruskal-Wallis test). (**C**) Mean (± range) number of amino acid (AA) substitutions per coding sequence from high frequency iSNVs at p5 in each bird species (*, crow p5 vs robin p5, *P* = 0.0429, Kruskal-Wallis test with Dunn’s correction). (**D**) Mean (± range) ratios of nonsynonymous to synonymous variants per site (*d*_*N*_ */d*_*S*_) (left) and amino acid diversity (right) from p0 and p5 for each WNV protein coding region. Left: * E protein, *P* = 0.0284; **, nonstructural protein 2A (NS2A), *P* = 0.0064; *, NS4B, *P* = 0.0175. *d*_*N*_ */d*_*S*_ was set at 1 for replicates without synonymous single nucleotide variants (SNVs) and 0 without nonsynonymous SNVs in the coding region. Right: *, E, *P* = 0.0284; *, NS4B, *P* = 0.0328, Kruskal-Wallis test. (**E**) High frequency nonsynonymous iSNVs from all bird passages were plotted according to their position in the WNV genome. Individual high frequency iSNVs can be found in S2 Table.

Analysis of reconstructed haplotypes that arose during passage and high frequency iSNVs (i.e. frequency > 0.02) was conducted to minimize the impact of differences in sequencing coverage and to assess positive selection. 0.02 was selected as a cutoff for “high frequency” mutations because it includes the top 5% of a gamma distribution of all VPhaser2-accepted iSNVs. The proportion of iSNVs that were high frequency after p5 was the greatest within robin-passaged WNV populations (16.5%) compared to sparrows (4.9%) and crows (4.8%) (Fig 4A). Reconstructed haplotypes from high frequency iSNVs were then used to assess the selective pressures that lead to haplotype replacement during passage (Fig 4B). The ancestral p0 virus population was composed of a single dominant haplotype that remained dominant after a single passage in all bird species. After p5, the ancestral haplotype remained dominant in crows, but not in sparrows and robins. Furthermore, high frequency iSNVs from crows contributed significantly fewer amino acid substitutions per coding sequence compared to robins after p5 (Fig 4C). Examination of *d*_*N*_ */d*_*S*_, amino acid diversity and high frequency nonsynonymous iSNVs across the WNV genome demonstrated that, in general, selection was the strongest in the structural protein coding regions (Fig 4D and 4E). Specifically, passage in robins imposed significant selective pressures on the envelope (E) protein coding region that heavily targeted ectodomains (ED) I and II. The apparent selection of the nonstructural protein 4B (NS4B) from sparrow passaging is the result of a single high frequency nonsynonymous iSNV (S2 Table). Individual high frequency iSNVs fluctuated in frequency through passaging and all nonsynonymous high frequency iSNVs were unique to its passage lineage (i.e. no “signature mutations” were detected that served as markers for replication in any particular bird species, see S2 Table).

### Interhost genetic divergence

**Fig 5:**
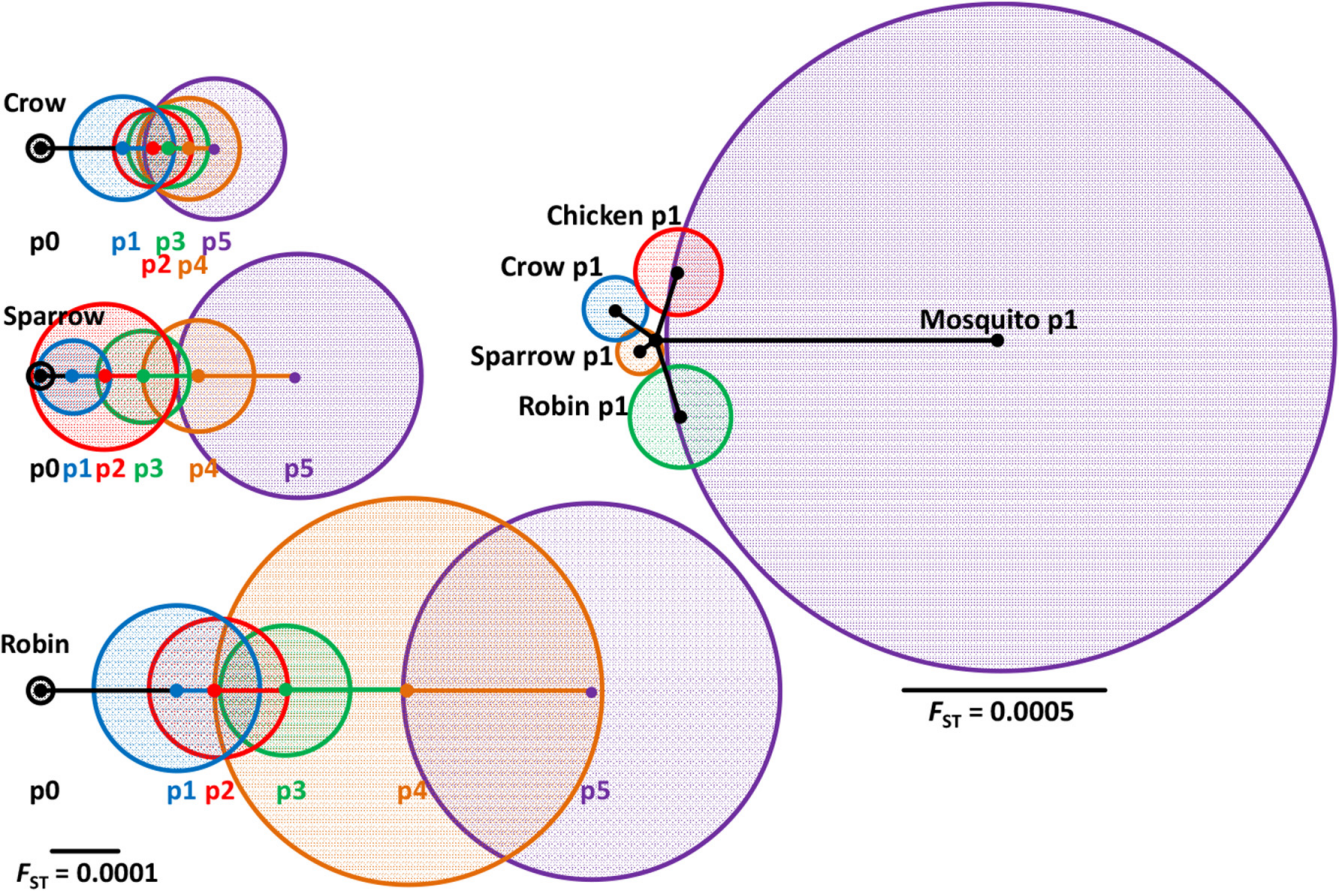
Differential interhost divergence of viral populations between individuals, sequential passage and host species. Circle diameters represent divergence (*F*_ST_) between individuals within a passage. Lines connecting circle centers represent between-passage divergence and were measured using combined replicates. *F*_ST_ from replicate means at p5 among crows (2 × 10^–4^), sparrows (4 × 10^–4^) and robins (6 × 10^–4^) were significantly different (*P* = 0.0500). *F*_ST_ was similar after a single passage in wild birds and chickens (~2 × 10^–4^) and significantly different from *F*_ST_ after a single mosquito passage (2 × 10^–3^, *P* = 0.0174, Kruskal-Wallis test).

The standardized variance in iSNV frequencies (*F*_ST_) was then estimated from the coding sequence to determine the degree of genetic divergence among replicates within a passage and between passages (Fig 5). Viral populations from robins were more divergent compared to those from crows and sparrows. *F*_ST_ from WNV passaged once in young chickens was similar to wild-caught birds, but WNV passaged once in mosquitoes was much more divergent. These results are supported by analysis of haplotypes (S3 Fig). The p0 haplotype was still dominant in chicken p1 populations with a small minority of haplotypes containing single iSNVs, similar to wild birds (Fig 4B). In mosquitoes the ancestral haplotype became a minority after a single passage.

## Discussion

### Virus passage and phenotypic assessment

We examined WNV genetic diversity during the course of passage in birds that experience varying mortality due to WNV infection to assess how different hosts influence virus population structure and fitness. Passage in each host was accomplished in three concurrent biological replicates in order to control for the impact of individual wild-caught birds that may vary in several ways that could impact virus replication. Titers during passage were highly variable between individuals. However, mean titers did not significantly change during the course of passage, indicating that replication competence was retained and that overt increases in competitive fitness were not selected through our passage strategy.

Wild-bird passaged virus was similar to unpassaged WNV in viremia production. Only when more sensitive *in vivo* competitive fitness assays (i.e. comparative replication of the passaged and reference WNV in the same host) were conducted were changes apparent. Note that our definition of fitness here is restricted to the specific competition environment (within the bird or mosquito) and does not consider the larger ecological fitness required for maintenance in a complex arbovirus transmission cycle. Passage in all birds resulted in significant competitive fitness gains during replication in chickens. Interestingly, the fitness gains were smallest after WNV was passaged in the host that experiences the most mortality (crows), and largest in the most disease-resistant avian host (robins). Fitness gains were far less clear when virus competition was measured in mosquitoes. A limitation to our mosquito studies is that competition was conducted via intrathoracic inoculation, which bypasses the midgut, a major physiological barrier in mosquitoes. Intrathoracic inoculation was used because the volume of blood available and the virus titers would have likely made oral infection highly inefficient. Importantly, our results on WNV replication and fitness are supported by previous observations [14] indicating that high fitness is maintained through purifying selection in vertebrates, and that no tradeoff occurs when the virus is re-introduced into mosquitoes. Moreover, replicative fitness increases occur during passage in ecologically relevant wild birds, and these gains occur in a species-specific manner.

### Patterns of intrahost mutational diversity and selective pressures

To investigate the viral genetic and population determinants of the observed fitness gains, we characterized WNV at each passage using NGS. Our data suggests that although the overall complexity of the virus population was similar among different bird species, its composition, and the selective pressures that produced it appear to be bird species-dependent. Interestingly, WNV replication in the most disease-susceptible bird species seems to be positively associated with the number of unique iSNVs (i.e. mutational tolerance) and negatively associated with iSNV frequency (i.e. strength of selection). This observation requires further investigation using additional resistant and susceptible birds, but may provide important insights into which bird species are most likely to drive virus evolution toward fitness gains. Our data thus far suggests that more disease resistant birds such as robins would be most likely to fill this role as long as they produce sufficiently high titers to infect mosquitoes.

In this study we used various neutrality tests to determine whether intrahost WNV populations from each bird species were evolving non-randomly through purifying selection. While these tests all measure slightly different aspects of genetic diversity, all clearly demonstrate purifying selection in birds. This result confirms previous studies of WNV passaged in young chickens [11], and indicates that our approaches to sequencing and analysis, although they differ significantly from those reported previously, produce results consistent with other methods.

Our studies also provide some evidence for positive selection during bird infection. We found that WNV passage in robins resulted in more amino acid substitutions that reach high frequency compared to crows. In addition, the ancestral haplotype tended to be displaced by novel mutants that arose during passage in sparrows and robins. These data suggest that positive selection within hosts is stronger in less susceptible bird species [26].

Examination of patterns of variation across the WNV genome provides additional evidence for differences in host selective environment. We found, consistent with previous reports on dengue virus populations [27], the highest variant frequencies in ectodomains I and II of the E coding sequence of WNV passaged in robins. The mechanisms that lead to the emergence of these variants are not currently clear. Although the E protein contains most neutralizing epitopes, the earliest neutralizing antibody responses observed in birds generally occur at around 5 to 7 days post infection [23,28]. Other mechanisms that could impact selection on the E protein include resistance to the early antiviral states induced by type I interferon [29,30] and alternate methods for virus entry and uncoating of the viral RNA [31]; though these mechanisms need further investigation, especially in birds. Our results suggest that in relatively resistant hosts, novel variants may rise to high frequency within the context of purifying selection. The notion that positive selection occurs in robins is further supported by our data showing that virus diverged most during replication in them. It is, however, balanced by a lack of evidence of a selective sweep, i.e. a rapid reduction in genetic diversity as a novel variant becomes very prominent in the population. Clearly further studies are needed to confirm whether and how positive selection contributes to WNV population structure in birds.

### Defective genomes

Compared to other RNA viruses, arboviruses have low long-term rates of amino acid substitution [32]. This is at least partially due to the fact that most mutations are deleterious because of evolutionary constraints on arbovirus genomes [33]. We provide evidence that accumulation of deleterious mutations, or defective viral genomes, is unequal between hosts; WNV populations replicating in wild-caught crows accumulate the most defective genomes, and WNV replicating in robins accumulate the least. Defective genomes are often found during laboratory and natural virus infections [17,34] and can persist through multiple rounds of transmission [35,36]. Using both bioassays (i.e. GE:PFU) and sequencing data (i.e. iLVs per coding sequence), we found that the accumulation of WNV defective genomes during infection was positively correlated with viral load. This apparent density-dependent selection of deleterious mutations likely occurs via functional complementation, which becomes more efficient as effective multiplicity of infection (MOI, i.e. intrahost viral load) increases [37,38]. In addition, high MOI environments tend to tolerate neutral mutations that can become deleterious in a new environment [39]. Taken together, these studies provide a framework to understand how WNV replication in high-viremic crows leads to a broader network of potentially deleterious mutations and limited selection for adaptive amino acid substitutions, especially when compared to WNV replication in robins. The rather modest fitness gains experienced by crow-passaged WNV support this observation.

### Conclusions

**Fig 6:**
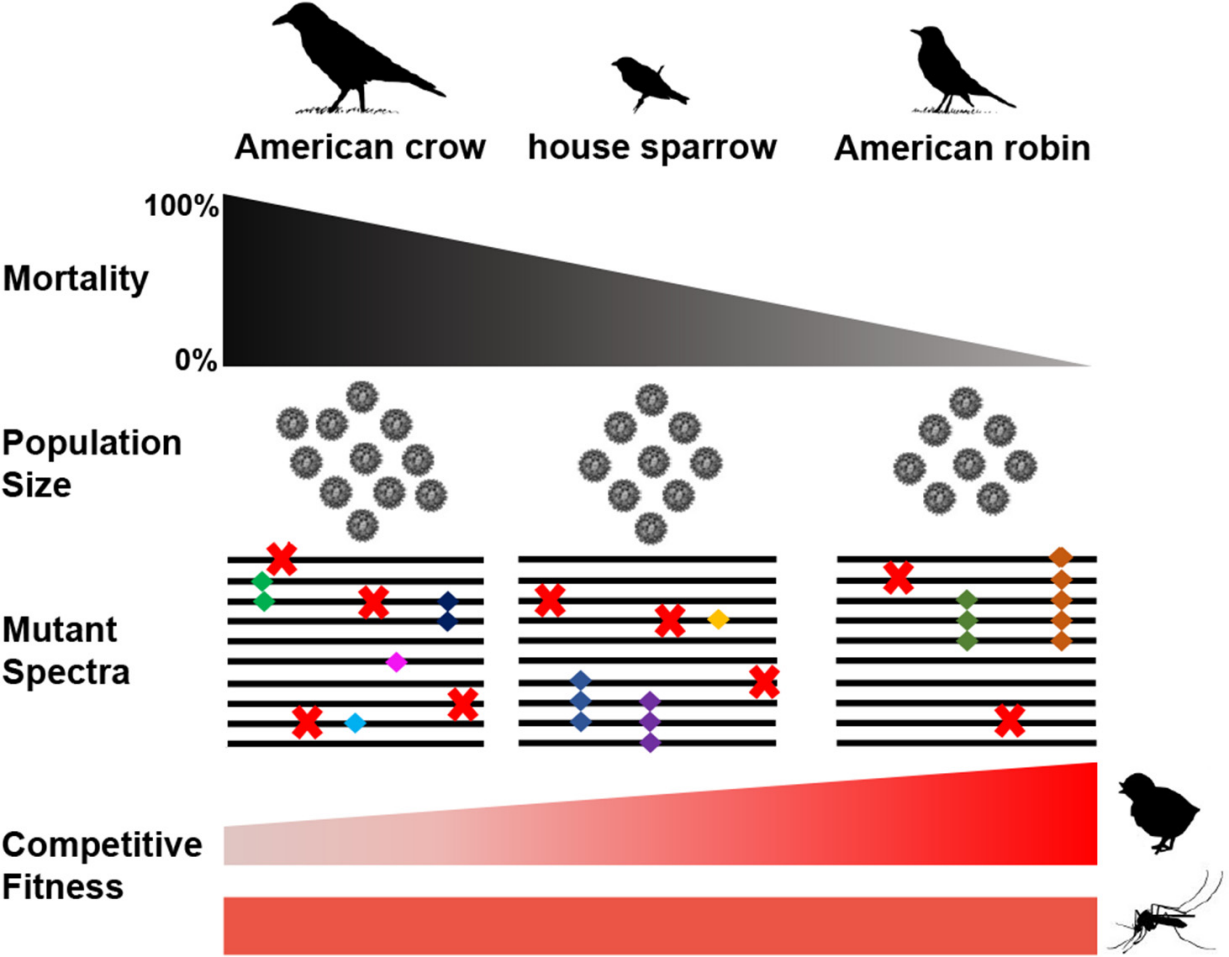
Species-specific composition of WNV populations and competitive fitness. Host mortality and intrahost WNV population sizes are associated with WNV population structure and competitive fitness. The WNV populations from all bird species contain ~1 mutation per genome. However in the crow environment, WNV populations are more tolerant of unique and deleterious mutations (e.g. insertions and deletions), but few mutations rise to high frequency. In the most disease-resistant bird species, robins, the WNV populations are under stronger selection pressures. Robin-associated WNV populations are less tolerant of unique and deleterious mutations, and more mutations reach high frequency. The selective environment of more disease-resistant birds was also positively associated with competitive fitness in young chickens, but not in mosquitoes. Population size: each “virus” represents a log_10_ of GE/ml. Mutant spectra: “X” represents deleterious mutations, “diamonds” represent neutral or advantageous mutations, and diamonds of the same color represents the same mutation.

The results presented here shed light on the selective forces that shape WNV populations in nature. We demonstrate that selective pressures that control WNV populations seem to occur in a species-specific manner (Fig 6). All three bird species evaluated have been suggested to be significant drivers of WNV outbreaks, with robins receiving particular attention due to findings indicating that this species is more frequently fed upon by mosquito vectors [24]. During intrahost WNV replication, our studies suggest that disease-susceptibility is positively associated with mutational tolerance and negatively associated with the strength of selection. This means that robins also may better maintain high fitness in WNV populations than do birds that are more susceptible to disease. While it is tempting to speculate that robins are significant generators of WNV genetic diversity, we also confirm herein that mosquitoes are much more efficient in generating mutational diversity in the WNV system. Moreover, these data suggest that intrahost virus evolutionary dynamics are associated with host resistance to disease in several ways and provide an important insight towards the genetic and ecological factors that influence RNA virus emergence.

## Materials and Methods

### Ethics statement

Wild birds were collected from under US Fish and Wildlife Service (#MB91672A-0) and Colorado Parks and Wildlife (#13TRb2106) permits and with permissions from landowners. No endangered or protected species were caught or harmed during the study. Experiments involving animals were conducted in accordance with protocols approved by the Colorado State University (CSU) Institutional Animal Care and Use Committee (#12-3694A) and the recommendations set forth in the Guide for the Care and Use of Laboratory Animals of the National Institutes of Health.

### Serial passage of WNV in wild-caught birds

A WNV infectious clone (WNVic) was previously constructed from an American crow kidney isolate collected during the 2000 outbreak in New York City [25,40]. The WNVic contains a naturally selected proline at amino acid site 249 in nonstructural protein 3 (NS3) allowing it to replicate to high titers in wild birds [18,41]. Wild birds were collected in Northern Colorado from 2013 to 2014 using mist nets (house sparrows and American robins) and cannon nets (American crows). All birds were bled prior to inoculation and serum was tested by plaque reduction neutralization test to confirm that all birds used for subsequent studies were WNV seronegative. The virus strain used to initiate the passage series was derived from a WNVic as previously described [25]. Virus was harvested from the supernatant of BHK cells transfected with linearized plasmid, stored at -80°C and used without further passage. Viruses were administered to birds by subcutaneous inoculation to the breast region with 1,000 WNV PFU/100 μl, a dose similar to mosquito transmission [42], in inoculation medium (endotoxin and cation-free phosphate buffered saline with 1% FBS). Birds were bled from the jugular vein at the time of peak viremia on 3 days post-infection (dpi). Serum was titered by standard plaque assay on African green monkey kidney cells (Vero, ATCC CCL-81) and stored at -80°C until used for subsequent passage or sequencing as described below. The first passage series utilized seven birds for each wild-caught species and the three birds with the median viral titers were used to start three independent replicate lineages, each including three naïve birds (i.e. replicates ‘a’, ‘b’, and ‘c’). From each group of three birds, the serum with the median viral titer was used to continue passaging to another cohort until five serial passages were completed. The WNVic derived virus was also passaged once in three young chickens for 3 dpi and two individual *Cx*. *quinquefasciatus* mosquitoes for 14 dpi to compare viral populations from commonly used laboratory vertebrate host and invertebrate vector models, respectively. See S1 Text for information about housing and care of wild-caught birds, chickens and mosquitoes.

### Phenotypic assessment

The infection phenotype of each WNV lineage after five passages (p5) in wild-caught birds was compared to the unpassaged (p0) WNV in the same bird species as virus passage, young chickens (two-days old), and *Cx*. *quinquefasciatus* mosquitoes (4–7 days post emergence). Viremia and survival was measured from birds were inoculated with 1,000 PFU of p5 or p0 WNV (n = 4–5 birds/virus) for up to 6 dpi. As defined here, competitive fitness compares the replication of a competitor virus (i.e. serial passaged p5 WNV) and a standard WNV reference (WNV-REF) during infection of the same host. Competitive fitness is quantified by the proportion of competitor to WNV-REF genotypes using sequence chromatograms (i.e. quantitative sequencing) [43]. WNV-REF was generated from an infectious clone as described above and in S1 Text and is indistinguishable from the parental virus in replication in cells and relevant organisms [44]. Competitive fitness assays of co-inoculated birds and mosquitoes with equally mixed WNV-REF and p5 competitor virus was conducted as described in S1 Text.

### Sequencing and data analysis

Virus libraries were prepared for RNA sequencing on the Illumina HiSeq 2000 platform (Beckman Coulter Genomics, Danvers, MA) using the NuGEN Ovation RNA-Seq System V2 and Ultralow Library kit (San Carlos, CA) (See SI Text for more details). Fastq files containing read data were demultiplexed using CASAVA and custom scripts that impose high stringency (0 mismatches) in the barcode region of each read. The sequence of the input WNV strain was determined from three independent biological sequencing replicates of the input virus using the Trinity assembler [45]. 100 nt paired-end reads were then aligned to this “input” sequence using MOSAIK [46]. Duplicate reads were removed using the MarkDuplicates tool within Picard to limit the influence of PCR artifacts and multiply sequenced clusters on variant calling with Vphaser2 [47]. Variants with significant strand bias were removed to reduce the potential for false-positives [48]. Variants called using Vphaser2 were used for subsequent data analysis unless otherwise specified. Analysis was limited to the protein coding sequences; and iSNVs and iLVs (includes both insertions and deletions) were analyzed separately.

Hamming distances from the p0 “input” virus were calculated for each population by dividing the total number of polymorphisms by the average coding sequencing coverage. Mean viral population complexity was calculated by the S_N_ at each site using the following equation [49]:

$$S_{N}{}_{=}-pi\left(Lnpi\right)+\left(1-pi\right)\left(Ln\left(1-pi\right)\right)/LnN$$

where *p* is the frequency of the iSNV at site *i* and *N* is the coverage at that site. At a single nucleotide position, a S_N_ score of 0 indicates a single nucleotide was present (i.e. no polymorphism) while a score of 1 represents maximum complexity (i.e. equal numbers of alternate nucleotides). The S_N_ at all protein coding sequence nucleotides loci were averaged to estimate the viral population complexity.

High frequency iSNVs were subjected to an additional analysis to reduce the possibility that conclusions drawn from the complete dataset were dependent on extremely rare variants. To establish a threshold for “high frequency” iSNVs, all of the Vphaser2 accepted variants detected in this study (n = 6052) were log_10_ transformed, increased by 3.75 (to make all of the values positive) and fit to a gamma distribution, where α = μ^2^/s^2^ and β = E[μ]/s^2^, using R (data did not fit a beta distribution). An iSNV frequency >0.02 was determined to be in the upper 5% of the gamma distribution and was used to define high frequency SNVs detected through WNV passage in birds (n = 341 individual SNVs). The sequencing reads from p0, p1 and p5 were aligned to the WNV genome using *mpileup* from the VarScan2 software package [50] and haplotypes were reconstructed using QuasiRecomb 1.2 [51] with the flags ‘-r 97–10395’, to reconstruct haplotypes from the entire coding sequence with respect to reference genome numbering, ‘-K 1–10’, to use a bigger interval of generators and ‘-noRecomb”, to disable the recombination process because it was not expected from the viral population and to reduce the runtime. To increase haplotype specificity, the flag ‘-conservative’ was employed and analysis was restricted to haplotypes containing high frequency SNVs (i.e. >0.02).

*pN* and *d*_*N*_ */d*_*S*_ were used to test for intrahost selection [33]. DnaSP (version 5) [52] was used to determine the number of nonsynonymous and synonymous sites to calculate *d*_*N*_ */d*_*S*_ using the Nei-Gojorori method [53] with the following modifications for NGS data. *N*_*d*_ and *S*_*d*_ (i.e. the numbers of detected nonsynonymous and synonymous mutations, respectively) were calculated for each viral population by the sum of individual nonsynonymous and synonymous VPhaser2 accepted iSNV frequencies and the passage consensus sequence was used to determine the number of nonsynonymous and synonymous sites. The number of nonsynonymous (7843.67) and synonymous (2455.33) sites in the ancestral p0 consensus sequence were used to determine that *pN* prior to selection is ~ 0.76. In addition, 50 most frequent haplotypes reconstructed from p1 and p5 from each bird species were analyzed using the Fu and Li’s *F* [54] and Fay and Wu’s *H* [55] statistical tests of neutrality in DnaSP with a window length of 100, a step size of 25 and the p0 consensus sequence as an outgroup to infer the ancestral nucleotide state.

*F*_ST_ was used to estimate the extent of interhost genetic divergence using a scale between 0 and 1, and the extent of *F*_ST_ change between populations represents the degree of genetic divergence. Specifically, in-house FORTAN scripts were used to calculate *F*_ST_ using equations 1, 2 and 4 by Fumagalli et al. [56]. Intrahost SNV frequencies determined by *mpileup* and *readcounts* from the VarScan2 software package [50] were used to estimate the per site heterozygosity in biological replicates compared to the total population (e.g. all biological replicates within passage) at a single passage (i.e. intra-passage) and the per site heterozygosity between passage replicates (i.e. inter-passage).

For estimation of the probability of resampling for the iLV data, we used the phyper command in R (www.R-project.org). We calculated that a total of 51,490 single nucleotide iLVs were possible by multiplying the length of the coding sequence (10,299 nt) by the 5 different kinds of iLVs that could occur at each site (one deletion and four different nt insertions). We then used phyper to obtain the probability of sampling overlap of 400 iLVs out of 600 sampled (reflecting a reasonable approximation of our observed data for crows) given that 51,490 iLVs are possible. Simulation studies were conducted in R by randomly sampling 600 individuals, with replacement, from a set of 51,490 and comparing the sets. T-tests, Kruskal Wallis tests, and correlation statistics were obtained using R and GraphPad Prism (La Jolla, CA).

## Supporting Information

S1 Fig Phenotypic analysis in wild-caught birds and bloodfed mosquitoes. (**A**) Wild-caught crows infected with crow-passaged WNV (n = 4 each) were assessed daily for viremia production (represented as the mean ± SD of WNV plaque forming units [pfu]/ml of serum) and survival were compared by two-way ANOVAs with Tukey’s corrections for multiple comparisons. Left: *, crow-passage 5 replicate “b” (p5b) vs p0 at 2, 3 and 4 days post infection (dpi), *P* = 0.0206, 0.0382 and 0.0185, respectively. Right inset: *, p5b vs p0, *P* = 0.0309. However, this significance is likely due to lower than expected WNVic p0 virus titers (100× lower at 3 dpi than the first passage of WNVic shown in Fig 1). Crow passages p5a and p5c did not lead to significant differences in viremia and survival. Due to decreasing sample sizes caused by mortality, analysis was limited to 1–4 dpi. The dashed lines indicate the assay detection limits. (**B**) Wild-caught sparrows infected with sparrow-passaged WNV were assessed as described in (A) and led to no significant differences in viremia and survival compared to p0 viruses (n = 4 each). (**C**) WNV competitive fitness was measured by the change of mean proportion of the competitor (i.e. p5 WNV) from the inocula compared to after 3 days post infection (dpi) in crows and sparrows by unpaired *t*-tests (crows, *P* = 0.0017; sparrows *P* <0.0001). The biological replicates are shown as magenta squares (replicate ‘a’), yellow triangles (replicate ‘b’) and teal circles (replicate ‘c’). (**D**) Crow p5 WNV competitive fitness in *Culex quinqefasciatus* mosquitoes 14 dpi by oral inoculation was determined as described in (C) (bodies, *P* = 0.0258; legs/wings and saliva, not significant).
(TIF)

S2 Fig Sequencing coverage of the virus genome is correlated with intrahost virus population sizes. (**A**) WNV sequencing coverage plotted by genome position for the input virus used to initiate passaging (sequenced as technical replicates, p0a-p0c) and after five passages in wild-caught crows, sparrow and robin viruses (sequenced as biological replicates, p5a-p5c). (**B**) Intrahost virus population sizes measured by genome equivalents (GE)/ml of bird serum after each sequential passage. (**C**) Correlations of sequencing coverage of the WNV genome to the intra-host virus population sizes from each bird species using individuals were made by the Pearson’s correlation coefficient (crows, r = 0.5249, *P* = 0.0445; sparrows, r = 0.9145, *P* = <0.0001; robins r = 0.7041, *P* = 0.0034).
(TIF)

S3 Fig Haplotype reconstruction of viral populations passaged in chickens and mosquitoes. Haplotypes were reconstructed from the high frequency iSNVs (i.e. > 0.02) from input WNV and after one passage in young chickens and *Culex quinquefasciatus* mosquitoes and are represented by the number of single nucleotide variants (SNVs) per haplotype (Hamming distance from the p0 haplotype).
(TIF)

S1 Table Summary of iSNVs from next generation sequencing data. (DOCX)

S2 Table List of high frequency iSNVs detected during passaging. (DOCX)

S1 Text Additional methods used in this study. (DOCX)
