# Supplementary material for: Experimental Evolution of an RNA Virus in Wild Birds: Evidence for Host-Dependent Impacts on Population Structure and Competitive Fitness
Source: PLoS Pathog. 2015 May 20;11(5):e1004874. doi: 10.1371/journal.ppat.1004874 (PMC4439088; doi:10.1371/journal.ppat.1004874)
Supplement: S1 Table — (DOCX) [file ppat.1004874.s004.docx]

| **Table S1.** Summary of West Nile virus intrahost single nucleotide variant (iSNV) data from next generation sequencing through five passages in wild-caught American crows, house sparrows and American robins and one passage in young chickens and *Culex quinqefasciatus* mosquitoes. | | | | | | | |
| --- | --- | --- | --- | --- | --- | --- | --- |
| **Passage series/replicate** | **Average genome coverage** | **iSNV sites** | **Total iSNVs detected** | **iSNV rate** | **iSNVs/coding sequence** | **Average iSNV frequency** | **Amino acid substitutions/coding sequence** |
| p0a | 36,220 | 104 | 6,007 | 1.61E-05 | 0.166 | 0.0016 | 0.141 |
| p0b | 34,753 | 99 | 6,615 | 1.85E-05 | 0.190 | 0.0019 | 0.155 |
| p0c | 36,203 | 83 | 5,057 | 1.36E-05 | 0.140 | 0.0017 | 0.118 |
| crow p1a | 37,114 | 140 | 33,811 | 8.85E-05 | 0.911 | 0.0067 | 0.737 |
| crow p1b | 47,285 | 115 | 10,347 | 2.12E-05 | 0.219 | 0.0019 | 0.177 |
| crow p1c | 44,889 | 162 | 13,699 | 2.96E-05 | 0.305 | 0.0020 | 0.242 |
| crow p2a | 43,505 | 147 | 30,254 | 6.75E-05 | 0.695 | 0.0049 | 0.505 |
| crow p2b | 53,178 | 137 | 30,601 | 5.59E-05 | 0.575 | 0.0041 | 0.191 |
| crow p2c | 44,097 | 151 | 16,512 | 3.64E-05 | 0.374 | 0.0027 | 0.254 |
| crow p3a | 44,806 | 137 | 16,892 | 3.66E-05 | 0.377 | 0.0028 | 0.272 |
| crow p3b | 46,837 | 173 | 38,542 | 7.99E-05 | 0.823 | 0.0048 | 0.256 |
| crow p3c | 38,480 | 170 | 16,571 | 4.18E-05 | 0.431 | 0.0027 | 0.298 |
| crow p4a | 41,191 | 167 | 21,315 | 5.02E-05 | 0.517 | 0.0031 | 0.276 |
| crow p4b | 34,882 | 124 | 37,045 | 1.03E-04 | 1.062 | 0.0089 | 0.210 |
| crow p4c | 43,308 | 174 | 27,175 | 6.09E-05 | 0.627 | 0.0036 | 0.226 |
| crow p5a | 44,023 | 212 | 36,926 | 8.14E-05 | 0.839 | 0.0040 | 0.403 |
| crow p5b | 33,257 | 181 | 45,482 | 1.33E-04 | 1.368 | 0.0080 | 0.274 |
| crow p5c | 36,869 | 154 | 28,737 | 7.57E-05 | 0.779 | 0.0050 | 0.280 |
| sparrow p1a | 43,878 | 168 | 34,544 | 7.64E-05 | 0.787 | 0.0049 | 0.276 |
| sparrow p1b | 44,277 | 127 | 14,852 | 3.26E-05 | 0.335 | 0.0027 | 0.177 |
| sparrow p1c | 28,817 | 84 | 11,468 | 3.86E-05 | 0.398 | 0.0049 | 0.181 |
| sparrow p2a | 16,212 | 70 | 22,696 | 1.36E-04 | 1.400 | 0.0216 | 0.302 |
| sparrow p2b | 42,209 | 155 | 15,231 | 3.50E-05 | 0.361 | 0.0023 | 0.221 |
| sparrow p2c | 33,666 | 125 | 25,143 | 7.25E-05 | 0.747 | 0.0064 | 0.428 |
| sparrow p3a | 1,287 | 34 | 1,077 | 8.12E-05 | 0.837 | 0.0270 | 0.508 |
| sparrow p3b | 38,902 | 140 | 19,757 | 4.93E-05 | 0.508 | 0.0036 | 0.314 |
| sparrow p3c | 58,358 | 169 | 30,614 | 5.09E-05 | 0.525 | 0.0031 | 0.298 |
| sparrow p4a | 44,678 | 141 | 25,726 | 5.59E-05 | 0.576 | 0.0040 | 0.234 |
| sparrow p4b | 38,691 | 150 | 14,818 | 3.72E-05 | 0.383 | 0.0025 | 0.281 |
| sparrow p4c | 28,312 | 99 | 16,489 | 5.66E-05 | 0.582 | 0.0060 | 0.235 |
| sparrow p5a | 32,529 | 121 | 126,348 | 3.77E-04 | 3.884 | 0.0332 | 0.856 |
| sparrow p5b | 29,719 | 80 | 9,077 | 2.97E-05 | 0.305 | 0.0038 | 0.183 |
| sparrow p5c | 39,358 | 108 | 45,526 | 1.12E-04 | 1.157 | 0.0108 | 0.196 |
| robin p1a | 7,994 | 78 | 4,132 | 5.02E-05 | 0.517 | 0.0073 | 0.397 |
| robin p1b | 29,136 | 143 | 10,650 | 3.55E-05 | 0.366 | 0.0026 | 0.293 |
| robin p1c | 9,648 | 46 | 1,916 | 1.93E-05 | 0.199 | 0.0047 | 0.124 |
| robin p2a | 11,022 | 39 | 5,007 | 4.41E-05 | 0.454 | 0.0149 | 0.332 |
| robin p2b | 26,211 | 206 | 17,449 | 6.46E-05 | 0.666 | 0.0034 | 0.497 |
| robin p2c | 35,058 | 192 | 20,276 | 5.62E-05 | 0.578 | 0.0030 | 0.394 |
| robin p3a | 25,759 | 112 | 22,606 | 8.52E-05 | 0.878 | 0.0081 | 0.499 |
| robin p3b | 3,442 | 75 | 3,038 | 8.57E-05 | 0.883 | 0.0121 | 0.631 |
| robin p3c | 20,326 | 140 | 13,269 | 6.34E-05 | 0.653 | 0.0048 | 0.306 |
| robin p4a | 13,837 | 87 | 15,499 | 1.09E-04 | 1.120 | 0.0146 | 0.563 |
| robin p4b | 117 | 4 | 21 | 1.74E-05 | 0.179 | 0.0456 | 0.119 |
| robin p4c | 3,601 | 31 | 1,896 | 5.11E-05 | 0.527 | 0.0193 | 0.180 |
| robin p5a | 11,057 | 79 | 16,127 | 1.42E-04 | 1.459 | 0.0216 | 0.754 |
| robin p5b | 3,673 | 22 | 2,026 | 5.35E-05 | 0.552 | 0.0273 | 0.293 |
| robin p5c | 14,776 | 57 | 30,544 | 2.01E-04 | 2.067 | 0.0483 | 0.696 |
| Chicken p1a | 11,075 | 47 | 3609 | 3.16E-05 | 0.326 | 0.0085 | 0.150 |
| Chicken p1b | 17,087 | 140 | 13189 | 7.49E-05 | 0.772 | 0.0052 | 0.348 |
| Chicken p1c | 21,801 | 56 | 3626.00 | 1.62E-05 | 0.166 | 0.0033 | 0.107 |
| Mosquito p1a | 790 | 5 | 1659.00 | 2.04E-04 | 2.101 | 0.2889 | 2.071 |
| Mosquito p1b | 1,919 | 16 | 3300 | 1.67E-04 | 1.719 | 0.1119 | 1.598 |
