# Supplementary material for: Experimental Evolution of an RNA Virus in Wild Birds: Evidence for Host-Dependent Impacts on Population Structure and Competitive Fitness
Source: PLoS Pathog. 2015 May 20;11(5):e1004874. doi: 10.1371/journal.ppat.1004874 (PMC4439088; doi:10.1371/journal.ppat.1004874)
Supplement: S2 Table — (DOCX) [file ppat.1004874.s005.docx]

| **Table S2.** Characterization of high frequency single nucleotide variants, defined as having an intrahost frequency greater than 2%, that arose during West Nile virus passaging in wild-caught birds, young chickens and *Culex quinquefasciatus* mosquitoes. | | | | | | | | |  |
| --- | --- | --- | --- | --- | --- | --- | --- | --- | --- |
| **Site** | **Coding region** | **Nucleotide change** | **Ts/Tv** | **Codon position** | **S/N** | **Amino acid change** | **Highest**  **frequency** | **Passage of highest frequency** |  |
| 101 | C | CT | Ts | 2 | N | S2F | 0.225 | sparrpw p2c^a^ |  |
| 111 | C | AT | Tv | 3 | S |  | 0.190 | sparrow p1a |  |
| 148 | C | AG | Ts | 1 | N | K18E | 0.022 | crow p2c |  |
| 202 | C | AT | Tv | 1 | N | S36C | 0.024 | robin p2c |  |
| 340 | C | CT | Ts | 1 | S |  | 0.042 | robin p4b |  |
| 369 | C | GA | Ts | 3 | S |  | 0.715 | sparrow p5a |  |
| 407 | C | AG | Ts | 2 | N | K104R | 0.042 | robin p4b |  |
| 432 | C | AG | Ts | 3 | S |  | 0.020 | sparrow p4b |  |
| 539 | prM | TC | Ts | 2 | N | I25T | 0.020 | robin p3b* |  |
| 547 | prM | GA | Ts | 1 | N | A28T | 0.020 | robin p5a |  |
| 683 | prM | CT | Ts | 3 | N | A73V | 0.030 | crow p2c |  |
| 779 | prM | CT | Ts | 2 | N | A105V | 0.023 | crow p5a |  |
| 818 | prM | CT | Ts | 3 | N | T118I | 0.036 | crow p3c |  |
| 925 | prM | GA | Ts | 1 | N | V154M | 0.021 | crow p5a |  |
| 945 | prM | TC | Ts | 3 | S |  | 0.077 | robin p5a |  |
| 951 | prM | GA | Ts | 3 | S |  | 0.032 | crow p5b |  |
| 997 | E | TC | Ts | 1 | N | F11L | 0.028 | robin p3b |  |
| 1032 | E | TA | Tv | 3 | N | D22E | 0.049 | mosquito p1a |  |
| 1071 | E | TC | Ts | 3 | S |  | 0.832 | robin p5c |  |
| 1102 | E | AG | Ts | 1 | N | M46V | 0.293 | robin p5a |  |
| 1117 | E | GA | Ts | 1 | N | A51T | 0.088 | robin p5a |  |
| 1170 | E | CT | Ts | 3 | S |  | 0.032 | crow p4b |  |
| 1179 | E | AG | Ts | 3 | S |  | 0.077 | crow p1a |  |
| 1205 | E | CT | Ts | 2 | N | A80V | 0.191 | robin p5b |  |
| 1238 | E | TC | Ts | 2 | N | V91A | 0.023 | mosquito p1b |  |
| 1272 | E | CT | Ts | 3 | S |  | 0.046 | chicken p1a |  |
| 1395 | E | CT | Ts | 3 | S |  | 0.101 | sparrpw p4c |  |
| 1440 | E | GT | Tv | 3 | N | Q158H | 0.037 | robin p3b |  |
| 1483 | E | GA | Ts | 1 | N | A173T | 0.031 | sparrow p3a |  |
| 1491 | E | AG | Ts | 3 | S |  | 0.020 | sparrpw p1c |  |
| 1496 | E | CT | Ts | 2 | N | T177I | 0.043 | robin p3c |  |
| 1545 | E | GA | Ts | 3 | S |  | 0.728 | sparrow p5a |  |
| 1557 | E | TC | Ts | 3 | S |  | 0.672 | sparrow p2a |  |
| 1567 | E | TC | Ts | 1 | N | Y201H | 0.056 | robin p2c |  |
| 1569 | E | CT | Ts | 3 | S |  | 0.357 | crow p4b |  |
| 1661 | E | TC | Ts | 2 | N | V232A | 0.028 | robin p4c |  |
| 1701 | E | AG | Tv | 3 | S |  | 0.027 | robin p4c |  |
| 1893 | E | CT | Ts | 3 | S |  | 0.023 | crow p5b |  |
| 1938 | E | CT | Ts | 3 | S |  | 0.097 | sparrpw p4c |  |
| 1974 | E | TC | Ts | 3 | S |  | 0.160 | chicken p1b |  |
| 2007 | E | CT | Ts | 3 | S |  | 0.048 | crow p5c |  |
| 2029 | E | TC | Ts | 1 | S |  | 0.021 | chicken p1a |  |
| 2077 | E | GT | Tv | 1 | N | V371F | 0.036 | robin p3b |  |
| 2114 | E | AT | Tv | 2 | N | Y383F | 0.024 | chick p1a |  |
| 2152 | E | CT | Ts | 1 | N | H396Y | 0.020 | sparrpw p2c |  |
| 2202 | E | CT | Ts | 3 | S |  | 0.022 | sparrpw p3c |  |
| 2321 | E | TC | Ts | 2 | N | L452S | 0.032 | crow p5a |  |
| 2345 | E | TC | Ts | 2 | N | I460T | 0.041 | robin p3b |  |
| 2346 | E | AG | Tv | 3 | N | I460M | 0.814 | robin p5c |  |
| 2518 | NS1 | AC | Tv | 1 | N | S17R | 0.515 | crow p1a |  |
| 2601 | NS1 | GA | Ts | 3 | S |  | 0.023 | sparrow p3b |  |
| 2607 | NS1 | TC | Ts | 3 | S |  | 0.058 | robin p3c |  |
| 2648 | NS1 | TC | Ts | 2 | N | V60A | 0.030 | robin p3b |  |
| 2679 | NS1 | AT | Tv | 3 | S |  | 0.035 | crow p2a |  |
| 2688 | NS1 | CA | Tv | 3 | N | D73E | 0.032 | sparrpw p1c* |  |
| 2734 | NS1 | GT | Tv | 1 | N | V89L | 0.067 | robin p3b |  |
| 2833 | NS1 | AG | Ts | 1 | N | I122V | 0.054 | robin p5a |  |
| 2856 | NS1 | CT | Ts | 3 | S |  | 0.151 | sparrpw p5c |  |
| 3123 | NS1 | TG | Tv | 3 | S |  | 0.020 | sparrow p3a |  |
| 3156 | NS1 | TC | Ts | 3 | S |  | 0.028 | robin p4a |  |
| 3162 | NS1 | GA | Ts | 3 | S |  | 0.047 | crow p5b |  |
| **Table S2 continued.** | | | | | | | | |  |
| **Site** | **Coding region** | **Nucleotide change** | **Ts/Tv** | **Codon position** | **S/N** | **Amino acid change** | **Highest**  **frequency** | **Passage of highest frequency** |  |
| 3186 | NS1 | TC | Ts | 3 | S |  | 0.034 | robin p3b |  |
| 3219 | NS1 | AC | Tv | 3 | S |  | 0.054 | mosquito p1a |  |
| 3243 | NS1 | TC | Ts | 3 | S |  | 0.033 | mosquito p1b^b^* |  |
| 3300 | NS1 | CT | Ts | 3 | S |  | 0.023 | crow p5c |  |
| 3306 | NS1 | CT | Ts | 3 | S |  | 0.037 | crow p2c |  |
| 3318 | NS1 | TC | Ts | 3 | S |  | 0.029 | robin p5a |  |
| 3327 | NS1 | CT | Ts | 3 | S |  | 0.119 | robin p5a^c^ |  |
| 3340 | NS1 | TG | Tv | 1 | N | C291G | 0.048 | robin p4c |  |
| 3467 | NS1 | TC | Ts | 2 | N | M333T | 0.030 | mosquito p1b |  |
| 3555 | NS2A | GA | Ts | 3 | S |  | 0.056 | crow p5b |  |
| 3561 | NS2A | CT | Ts | 3 | S |  | 0.117 | robin p5a |  |
| 3684 | NS2A | TC | Ts | 3 | S |  | 0.027 | sparrow p3a^d^ |  |
| 3749 | NS2A | TC | Ts | 2 | N | V75A | 0.047 | sparrow p4b |  |
| 3819 | NS2A | AG | Ts | 3 | S |  | 0.027 | sparrow p3b |  |
| 3852 | NS2A | GA | Ts | 3 | S |  | 0.039 | crow p4b |  |
| 3861 | NS2A | TC | Ts | 3 | S |  | 0.042 | robin p5a |  |
| 3893 | NS2A | AG | Ts | 2 | N | Q123R | 0.080 | sparrow p3b^e^ |  |
| 3899 | NS2A | TC | Ts | 2 | N | L125P | 0.056 | sparrow p3a |  |
| 3952 | NS2A | CT | Ts | 1 | S |  | 0.832 | robin p5c |  |
| 3997 | NS2A | CT | Ts | 1 | S |  | 0.048 | mosquito p1b |  |
| 4110 | NS2A | AG | Ts | 3 | S |  | 0.139 | crow p5b |  |
| 4221 | NS2B | AG | Ts | 3 | S |  | 0.129 | robin p5c |  |
| 4230 | NS2B | AG | Ts | 3 | S |  | 0.030 | crow p4b |  |
| 4318 | NS2B | AG | Ts | 1 | N | T34A | 0.026 | sparrpw p2c |  |
| 4332 | NS2B | CT | Ts | 3 | S |  | 0.021 | mosquito p1b |  |
| 4340 | NS2B | CT | Ts | 2 | N | A41V | 0.027 | sparrow p3a |  |
| 4392 | NS2B | GA | Ts | 3 | S |  | 0.037 | sparrpw p2c |  |
| 4484 | NS2B | AG | Ts | 2 | N | N89S | 0.020 | crow p5b |  |
| 4527 | NS2B | CT | Ts | 3 | S |  | 0.195 | robin p5b |  |
| 4530 | NS2B | TC | Ts | 3 | S |  | 0.719 | sparrow p5a |  |
| 4573 | NS2B | GA | Ts | 1 | N | V119I | 0.116 | robin p5a |  |
| 4596 | NS2B | CT | Ts | 3 | S |  | 0.054 | crow p3b |  |
| 4668 | NS3 | CT | Ts | 3 | S |  | 0.032 | chicken p1a |  |
| 4704 | NS3 | CT | Ts | 3 | S |  | 0.020 | crow p4c |  |
| 4749 | NS3 | CT | Ts | 3 | S |  | 0.086 | chick p1a |  |
| 4813 | NS3 | TC | Ts | 1 | N | Y68H | 0.027 | mosquito p1a |  |
| 4998 | NS3 | CT | Ts | 3 | S |  | 0.184 | robin p3a |  |
| 5002 | NS3 | CT | Ts | 1 | N | P131S | 0.020 | sparrpw p3c |  |
| 5136 | NS3 | CT | Ts | 3 | S |  | 0.023 | crow p5b |  |
| 5157 | NS3 | GT | Tv | 3 | N | E182D | 0.041 | robin p1b |  |
| 5184 | NS3 | AG | Ts | 3 | S |  | 0.025 | robin p2c |  |
| 5240 | NS3 | AG | Ts | 2 | N | K210R | 0.025 | robin p2a |  |
| 5469 | NS3 | GA | Ts | 3 | S |  | 0.023 | crow p5b |  |
| 5472 | NS3 | TC | Ts | 3 | S |  | 0.497 | sparrpw p5c |  |
| 5629 | NS3 | AG | Ts | 1 | N | I340V | 0.028 | robin p3b |  |
| 5668 | NS3 | AT | Tv | 1 | N | T353S | 0.020 | robin p1a |  |
| 5801 | NS3 | AG | Ts | 2 | N | K397R | 0.023 | chicken p1a |  |
| 5816 | NS3 | AT | Tv | 2 | N | D402V | 0.541 | mosquito p1b |  |
| 5874 | NS3 | CT | Ts | 3 | S |  | 0.034 | crow p4b |  |
| 5889 | NS3 | CT | Ts | 3 | S |  | 0.020 | crow p4c |  |
| 6393 | NS3 | GT | Tv | 3 | S |  | 0.032 | sparrpw p1c |  |
| 6410 | NS3 | GT | Tv | 2 | N | R600M | 0.027 | robin p3b |  |
| 6420 | NS3 | GA | Ts | 2 | S |  | 0.039 | robin p5c |  |
| 6481 | NS4A | CT | Ts | 1 | N | L5F | 0.038 | robin p5a* |  |
| 6543 | NS4A | CT | Ts | 3 | S |  | 0.125 | robin p5a |  |
| 6644 | NS4A | TC | Ts | 2 | N | L59S | 0.025 | crow p5a |  |
| 6672 | NS4A | CT | Ts | 3 | S |  | 0.063 | crow p5b |  |
| 6711 | NS4A | TA | Tv | 3 | S |  | 0.133 | sparrow p4a |  |
| 6734 | NS4A | TC | Ts | 2 | N | V89A | 0.641 | mosquito p1a |  |
| 6780 | NS4A | CT | Ts | 3 | S |  | 0.047 | crow p4b |  |
| 6849 | NS4A | GA | Ts | 3 | S |  | 0.033 | sparrpw p4c |  |
| 6871 | 2K | GA | Ts | 1 | N | V13M | 0.139 | sparrow p2a |  |

| **Table S2 continued.** | | | | | | | | | |
| --- | --- | --- | --- | --- | --- | --- | --- | --- | --- |
| **Site** | **Coding region** | **Nucleotide change** | **Ts/Tv** | **Codon position** | **S/N** | | **Amino acid change** | **Highest**  **frequency** | **Passage of highest frequency** |
| 6879 | 2K | GA | Ts | 3 | S | |  | 0.043 | sparrpw p1c |
| 7095 | NS4B | CT | Ts | 3 | S | |  | 0.027 | robin p4c* |
| 7236 | NS4B | CT | Ts | 3 | S | |  | 0.025 | robin p5a |
| 7320 | NS4B | CT | Ts | 3 | S | |  | 0.125 | robin p5a |
| 7362 | NS4B | TC | Ts | 3 | S | |  | 0.022 | sparrpw p2c |
| 7648 | NS4B | AT | Tv | 1 | N | | I245L | 0.718 | sparrow p5a |
| 7754 | NS5 | CT | Ts | 2 | N | |  | 1.000 | mosquito p1b |
| 7825 | NS5 | GA | Ts | 1 | N | | A49I | 0.048 | robin p4b |
| 7826 | NS5 | CT | Ts | 2 | N | | A49I | 0.048 | robin p4b |
| 8076 | NS5 | GA | Ts | 3 | S | |  | 0.027 | crow p1a |
| 8121 | NS5 | CT | Ts | 3 | S | |  | 0.042 | crow p5b |
| 8148 | NS5 | TC | Ts | 3 | S | |  | 0.023 | sparrow p3a |
| 8220 | NS5 | CT | Ts | 3 | S | |  | 0.123 | sparrpw p5c |
| 8261 | NS5 | AG | Ts | 2 | N | | K194R | 0.078 | robin p4c |
| 8289 | NS5 | GT | Tv | 3 | S | |  | 0.023 | robin p3b |
| 8499 | NS5 | CT | Ts | 3 | S | |  | 0.028 | robin p3b |
| 8553 | NS5 | TC | Ts | 3 | S | |  | 0.038 | crow p5c |
| 8679 | NS5 | AC | Tv | 3 | S | |  | 0.062 | sparrow p1b |
| 8883 | NS5 | CT | Ts | 3 | S | |  | 0.032 | crow p2c |
| 9039 | NS5 | TC | Ts | 3 | S | |  | 0.038 | crow p2a |
| 9054 | NS5 | AG | Ts | 3 | S | |  | 0.021 | robin p5a |
| 9086 | NS5 | AG | Ts | 2 | N | | K469R | 0.671 | mosquito p1a |
| 9136 | NS5 | CT | Ts | 1 | S | |  | 0.022 | robin p5a |
| 9285 | NS5 | TC | Ts | 3 | S | |  | 0.038 | chick p1c |
| 9704 | NS5 | TC | Ts | 2 | N | | L675P | 0.035 | sparrow p3a |
| 9730 | NS5 | CT | Ts | 1 | N | | H684Y | 0.030 | robin p1c |
| 9759 | NS5 | CT | Ts | 3 | S | |  | 0.026 | crow p5c |
| 9783 | NS5 | GA | Ts | 3 | S | |  | 0.033 | robin p2c |
| 10243 | NS5 | CT | Ts | 1 | S | |  | 0.681 | sparrow p5a |
| 10326 | NS5 | GA | Ts | 3 | S | |  | 0.089 | sparrow p5a |
| 10339 | NS5 | TC | Ts | 1 | N | | Y887H | 0.106 | robin p5b^f^ |
| 10375 | NS5 | TC | Ts | 1 | S | |  | 0.029 | sparrow p3a |
|  | | | | | |  |  |  |  |

C, capsid; prM, pre-membrane; E, envelope; NS, nonstructural; Ts, transition; Tv, transversion; N, nonsynonymous; S, synonymous.*, also detected in input p0 virus. ^a^, also detected in sparrow replicate “a”. ^b^, also detected in robin replicate “b”. ^c^, also detected in sparrow replicate “c”. ^d^, also detected in crow replicate “c”. ^e^, also detected in sparrow replicate “a”. ^f^, also detected in crow replicate “b”.
