# Supplementary material for: Experimental Evolution of an RNA Virus in Wild Birds: Evidence for Host-Dependent Impacts on Population Structure and Competitive Fitness
Source: PLoS Pathog. 2015 May 20;11(5):e1004874. doi: 10.1371/journal.ppat.1004874 (PMC4439088; doi:10.1371/journal.ppat.1004874)
Supplement: S1 Text — (DOCX) [file ppat.1004874.s006.docx]

**Supporting Materials and Methods**

**Housing and care of wild-caught Birds, chickens and mosquitoes**

Wild-caught birds were housed in 0.5 to 1m^3^ cages in groups of 3-4 with space for limited flight (sparrows and robins) and fed *ad libitum* water and a mixture of dry dog food (crows and robins), raisons (robins), earth worms (robins) and/or bird seed (sparrows) as described previously [[1-4](#_ENREF_1)]. Chickens (two-days old) were hatched from specific pathogen free eggs (Charles River Specific Pathogen Free Avian Services, Franklin, CT) and maintained as described previously [[5](#_ENREF_5)]. *Cx. quinquefasciatus* mosquitoes were reared from a long laboratory-established colony. Mosquitoes were maintained at 26-27°C and 70-80% relative humidity with a 16:8 L:D photoperiod. Water and 10% sucrose was provided ad libitum. Adult mosquitoes used for experiments were 4-7 days post-emergence. All animal infections were conducted within the Colorado State University ABSL-3.

**WNV-REF and competitive fitness assays**

The WNV-REF was created using site-directed mutagenesis [[6](#_ENREF_6)]. Five sequential synonymous changes were made to nucleotide positions 8313-8317 in the NS5 region of the genome, changing the parental sequence CTC TCA CGG to CTa agc aGG. The non-coding changes to WNV-REF did not affect the replication kinetics and infectivity compared to the WNVic [[7](#_ENREF_7)], making WNV-REF a useful standard to measure the fitness changes of WNVic after serial passage in birds [[7](#_ENREF_7),[8](#_ENREF_8)]. Birds were co-inoculated with 1000 PFU of equally mixed WNV-REF and p5 competitor virus (n = 4-5 birds/competition) and serum was collected 3 dpi as described above. *Cx. quinquefasciatus* mosquitoes were intrathoracically (IT) inoculated with 10 PFU/69 nl of equally mixed WNV-REF and p5 competitor virus (n = 40-60 mosquitoes/competition) using a Nanoject II (Drummond Scientific Company, Boomall, PA). Injected mosquitoes were held in quart-sized cardboard containers with water and 10% sucrose provided *ad libitum*. After 14 dpi, the mosquitoes were anesthetized with triethylamine and saliva was collected in capillary tubes for 30 minutes as previously described [[9](#_ENREF_9)]. Individual whole mosquito bodies were homogenized in 100 μl of cell culture medium (Eagle’s minimum essential medium, 10% heat-inactivated fetal bovine serum, 100 units/ml penicillin/streptomycin, 50μg/ml gentamicin, 1x L-glutamine and 125ng/ml fungizone) and a ball bearing using a Mixer Mill MM300 (Qiagen, Valencia, CA) for 30 s at 24 cycles/s. The homogenates were clarified by centrifugation for 5 min at 10,000 × g. Additionally, 6 log_10_ PFU/ml of crow p5 viruses mixed equally with WNV-REF was offered to mosquitoes in an infectious bloodmeal (n = 30-60 mosquitoes each). Mosquito midguts, legs/wings and saliva were collected from individual mosquitoes at 14 dpi and homogenized. Total RNA was isolated from 50 μl of bird serum, mosquito tissue homogenates and mosquito saliva using the Mag-Bind Viral DNA/RNA 96 kit (Omega Bio-Tek, Norcross, GA) on the KingFisher Flex Magnetic Particle Processor (Thermo Fisher Scientific, Waltham, MA ), according to manufacturer’s protocols, and RNA eluted in 50 μl of nuclease-free water.

**Library preparation**

Viral RNA was extracted from 50 μl of bird serum or mosquito body homogenate as described above with the addition of 1 μl of the RNase inhibitor SUPERase-In (Ambion, Austin, TX) and 0.5 μl of linear polyacrylamide (Ambion) added to the RNA followed by Turbo DNase treatment (Ambion). Total RNA was amplified using the NuGEN Ovation RNA-Seq System V2 (San Carlos, CA) and cDNA amplicons were sheared using the Covaris S2 Focused-ultrasonicator (Covaris, Woburn, MA) according to the manufacturer’s recommendations. Sequencing libraries were prepared from 22-100 ng of sheared cDNA using NuGEN’s Ovation Ultralow Library Kit according to the manufacturer’s recommendations. Agencourt RNAclean XP beads (Beckman Coulter Genomics, Pasadena, CA) were used for all purification steps. WNV GE/ml concentrations were quantified following 1) RNA extraction, 2) DNase treatment and 3) RNA-Seq cDNA synthesis steps by qRT-PCR using the iScript One-step RT-PCR Kit for probes (Bio-Rad Laboratories Inc., Hercules, CA) and a previously described probe and primer set [[10](#_ENREF_10)]. Briefly, 25 μl reactions were amplified using the CFX96 Real-Time instrument (Bio-Rad Laboratories Inc.), standard amplification conditions and WNV RNA standards prepared as previously described [[11](#_ENREF_11)]. Finished libraries were analyzed for correct size distribution using the Agilent Bioanalyzer High Sensitivity DNA chips (Agilent, Santa Clara, CA). Deep sequencing was performed using the Illumina HiSeq 2000 platform at Beckman Coulter Genomics (Danvers, MA) and 20-30 uniquely barcoded libraries were multiplexed per run.

**Supporting References**

1. Langevin SA, Bowen RA, Reisen WK, Andrade CC, Ramey WN, et al. (2014) Host Competence and Helicase Activity Differences Exhibited by West Nile Viral Variants Expressing NS3-249 Amino Acid Polymorphisms. Plos One 9.

2. Brault AC, Langevin SA, Bowen RA, Panella NA, Biggerstaff BJ, et al. (2004) Differential virulence of West Nile strains for American crows. Emerging Infectious Diseases 10: 2161-2168.

3. VanDalen KK, Hall JS, Clark L, McLean RG, Smeraski C (2013) West Nile virus infection in American Robins: new insights on dose response. Plos One 8: e68537.

4. Langevin SA, Brault AC, Panella NA, Bowen RA, Komar N (2005) Variation in virulence of West Nile virus strains for house sparrows (Passer domesticus). American Journal of Tropical Medicine and Hygiene 72: 99-102.

5. Jerzak GV, Bernard K, Kramer LD, Shi PY, Ebel GD (2007) The West Nile virus mutant spectrum is host-dependant and a determinant of mortality in mice. Virology 360: 469-476.

6. Lo MK, Tilgner M, Bernard KA, Shi PY (2003) Functional analysis of mosquito-borne flavivirus conserved sequence elements within 3' untranslated region of West Nile virus by use of a reporting replicon that differentiates between viral translation and RNA replication. J Virol 77: 10004-10014.

7. Fitzpatrick KA, Deardorff ER, Pesko K, Brackney DE, Zhang B, et al. (2010) Population variation of West Nile virus confers a host-specific fitness benefit in mosquitoes. Virology 404: 89-95.

8. Deardorff ER, Fitzpatrick KA, Jerzak GV, Shi PY, Kramer LD, et al. (2011) West Nile virus experimental evolution in vivo and the trade-off hypothesis. Plos Pathogens 7: e1002335.

9. Smith DR, Carrara AS, Aguilar PV, Weaver SC (2005) Evaluation of methods to assess transmission potential of Venezuelan equine encephalitis virus by mosquitoes and estimation of mosquito saliva titers. American Journal of Tropical Medicine and Hygiene 73: 33-39.

10. Lanciotti RS, Kerst AJ, Nasci RS, Godsey MS, Mitchell CJ, et al. (2000) Rapid detection of west nile virus from human clinical specimens, field-collected mosquitoes, and avian samples by a TaqMan reverse transcriptase-PCR assay. Journal of Clinical Microbiology 38: 4066-4071.

11. Brackney DE, Pesko KN, Brown IK, Deardorff ER, Kawatachi J, et al. (2011) West Nile virus genetic diversity is maintained during transmission by Culex pipiens quinquefasciatus mosquitoes. Plos One 6: e24466.
